# The emergence of social order in everyday interacting: re-conceptualizing a venerable sociological concept in light of conversation analysis

**DOI:** 10.3389/fsoc.2023.1102449

**Published:** 2023-11-27

**Authors:** Robert B. Arundale

**Affiliations:** University of Alaska Fairbanks, Fairbanks, AK, United States

**Keywords:** emergence, social order, interaction, conversation analysis, communication, sociology

## Abstract

For more than a century social theorists have asked how order at the macro-social level is related to human activity at the micro-social level. Among their answers are accounts of macro-level social order as emerging in micro-level relations among individuals. Sawyer’s account of macro-level emergence in micro-level interaction rests on the individual’s understandings of interactional frames. However, Rawls draws on Garfinkel and Sacks to argue that sociologist’s accounts of the macro-level interaction order need to be grounded in observable, micro-level social practices, instead of using conceptual abstractions like frames. Arundale’s Conjoint Co-constituting Model of Communicating is grounded in research on observable social practices in Conversation Analysis, and offers an account of the emergence, in particular episodes of everyday interacting, of properties that define micro-level social systems. That account provides the basis for an account of the emergence, in recurrent micro-level interacting over time and space, of properties that define macro-level social systems. The basic idea is not new: what is new is accounting for the emergence of macro-level social order in terms of the recurrent emergence of micro-level social order as participants engage observable social practices in everyday interacting. Re-conceptualizing the emergence of macro-social order addresses sociology’s longstanding puzzlement regarding the macro–micro link, and points to needed research.

## 1 Introduction

Since the early years of sociology as an independent discipline, social theorists have been beset with the question “What is the relationship between what is social and what is individual in human life?” or alternatively, “How is order at the macro social level related to order at the micro individual level?” Durkheim argued that “There can be no sociology unless societies exist, and … societies cannot exist if there are only individuals (Durkheim, [1897]1951, p. 38), adding that if only individuals exist, then “[s]ociological laws can be only a corollary of the more general laws of psychology; the ultimate explanation of collective life will consist in showing how it emanates from human nature in general” (Durkheim, [1895]1964, p. 98). Social theorists generally agree that both societies and individuals are real phenomena with empirical manifestations, and they have forwarded varied accounts of the relationship between them, ranging from conceptualizing them as a dichotomy in which underlying institutional structures shape the social actions of individuals, through conceptualizing social institutions as reducible to processes or states defined on individuals, to understanding the social order as emerging in on-going interacting among individuals.^1^

This chapter focuses on theories in the third category: those that seek to account for how, in Durkheim’s terms, collective social phenomena “emanate” from the general activities of individuals. Section 2 characterizes Sawyer’s (2005) examination of the history of sociological theorizing regarding emergence, in light of which he develops his account of emergence in everyday interaction: as individuals interact with one another, they construct and engage “interactional frames” that are emergent outcomes with causal power in shaping the direction of subsequent interaction. Section 3 examines Rawls (1987) argument, following Goffman’s lead, that the “interaction order” needs to be conceptualized as a social order *sui generis*, distinct both from institutional orders and from individual processes. But Rawls (1989, 2003) also argues that Goffman’s (1974) frame-based account of the interaction order is problematic because a “frame” is a theorist’s conceptual abstraction presumed to account for participant behavior, whereas drawing on Garfinkel’s (1967) and Sacks (1992) understandings of human interaction as rooted in participant’s concrete social practices would generate a more productive account of the interaction order. Sawyer’s (2005) frame-based account of social emergence is likewise problematic. Section 4 draws on research in Conversation Analysis, based in Garfinkel’s and in Sacks’ work, to develop an alternative account of the process by which interpretings of action and meaning evolve in everyday interacting among two or a few individuals: an account based directly in participant’s use of concrete social practices in everyday talk and conduct. This account is also an account of the emergence of properties that define two or a few participants as a micro-level complex system. That account serves in turn as the basis for re-conceptualizing macro-level social order as emerging as participants recurrently constitute actions and meanings across multiple episodes of everyday micro-level interacting, spread over time and across space. Section 5 returns to the opening questions regarding the emergence of social order in the activities of individuals.^2^

## 2 Sawyer’s theory of social emergence

In a series of publications beginning in Sawyer (2001, 2002a,b, 2003a,b,c, 2004) and culminating in Sawyer (2005), *Social Emergence: Societies as Complex Systems*, Sawyer develops his model of emergence against the background of what is arguably the most inclusive and careful examination of the convoluted history of sociological theorizing regarding the emergence of social order. Of special importance in this history is Durkheim’s early search for an explanation of how collective life emanates from the activities of individuals. A number of commentaries have found Durkheim’s approach wanting, especially as developed in Durkheim ([1895]1964) *The Rules of Sociological Method*, but Sawyer (2002a, 2005, Chap. 6) argues that revisiting Durkheim in view of late 20th century thinking on emergence makes apparent that he can be understood as an emergence theorist. Sawyer (2002a, p. 232) notes that “Durkheim never used the term ‘emergence’; rather, his phrase *sui generis* was used in a sense synonymous with contemporary uses of the term ‘emergent,’” and that “following common usage in the nineteenth century” he used “the terms ‘synthesis’ and ‘association’ when referring to emergent systemic phenomena that resulted from nonadditive combinations of elements.” Sawyer (2002a, pp. 244–5, 2005, p. 123) identifies a number of issues that Durkheim failed to resolve that prevented him from developing his “perspective into a full-fledged processual-dynamic view of social emergence” (Sawyer, 2005, p. 115), but adds that “[o]ne can hardly fault Durkheim for failing to resolve this complex and challenging issue, for it remains unresolved” (Sawyer, 2005, p. 116).

For Sawyer (2005, p. 6), a full-fledged, processual-dynamic account of emergence would be an account of “the nature of society as a complex system” that reveals the process and mechanism through which individuals in their relations with other individuals form “macro social phenomena, such as markets, the educational system, cultural beliefs, and shared social practices (e.g., politeness and power dynamics).” In developing his own account of social emergence, Sawyer utilizes research beginning in the 1990s on both complex systems and computer simulations of social institutions. *Complex systems* are physical and biological systems that are not just complicated, but that also exhibit not only properties that are non-linear, i.e., not predictable from initial conditions, but also properties that are non-additive. i.e., not the sum of a property of each of the system’s parts, but instead properties of the whole that are not exhibited by the parts of the system in isolation from one another. These non-linear, non-additive properties are the “emergent properties” that define the interconnected parts as a complex system. Living biological systems are not only complicated, multi-part, autonomous systems, but also complex systems exhibiting the key emergent property of life itself. A major disruption to almost any part of a living system, or of the connections between its parts, is very likely to terminate that key emergent property. In examining research on complex systems in general, and particularly research involving computer simulation of social emergence in artificial societies, Sawyer (2005, p. 166) argues that although “the question remains to what extent these models can be considered accurate representations of true human societies,” they nevertheless provide one means for addressing the question of how “macro-social phenomena emerge from individual action and then in turn constrain or otherwise influence future action?” (Sawyer, 2005, p. 162).^3^

Within this conceptual framework Sawyer provides a model of the process and mechanism of social emergence in human interaction that he labels the “Emergence Paradigm.” In his words:

In any social situation, there is a continuing dialectic: social emergence, where individuals are co-creating and co-maintaining ephemeral and stable emergents, and downward causation from those emergents. … During conversational encounters, interactional frames emerge, and these are collective social facts that can be characterized independently of individuals’ interpretations of them. Once a frame has emerged, it constrains the possibilities for action (Sawyer, 2005, p. 210). [E]ach action contributes to a continuing process of collaborative emergence at the same time that it is constrained by the shared emergent frame that exists at that moment. The emergent frame is a dynamic structure that changes with each action. No one can stop the encounter at any one point and identify with certainty what the frame’s structure is (Sawyer, 2005, 213).

For Sawyer (2005, pp. 214–5), then, “interactional frames” are central in explaining emergence in human interaction. More than 30 years earlier in *Frame Analysis* Goffman (1974, pp. 10–11) drew on Bateson’s (1972) concept of “frame” in noting that “I assume that definitions of a situation are built up in accordance with principles of organization which govern events—at least social ones—and our subjective involvement in them; frame is the word I use to refer to such of these basic elements as I am able to identify. That is my definition of frame.” Sawyer (2003b, 2005) does not explicitly define his concept of “frame,” nor does he cite Goffman’s detailed examination for purposes of comparison, further development, or critique. Sawyer (2003b) does provide examples of frames drawn from his extensive experience with improvisational (i.e., *sans* scripts or plots) theatre performances, but in the absence of explicit commentary, I find no indication that Sawyer defines “frame” in a way that differs from Goffman’s definition (cf. Verhoeven, 1985, p. 83).

For Sawyer, interactional frames include both ephemeral emergents and stable emergents. *Ephemeral emergents* are those that occur within a single encounter in the form of implicit (i.e., out of conscious awareness) metapragmatic features of language used by speakers “to reflexively communicate about the emergent process and flow of the encounter” (Sawyer, 2005, p. 182). In the context of improvisational theatre, interactional frames are what provides an actor with his or her definition of the situation at a given moment in the dialogue, or more colloquially, his or her sense of “what’s going on” and of his or her possible involvement in the activity. More specifically, the metapragmatic “interactional frame includes all of the pragmatic elements of a small-group encounter: the socially recognized roles and practices enacted by each participant, the publically shared and perceived motives of those individuals, the relationship among them, and the collective definition of the joint activity they are engaged in. The frame is constructed turn by turn: one person proposes a new development for the frame, and others respond by modifying or embellishing the proposal” (Sawyer, 2005, p. 182). *Stable emergents*, in contrast, are those that last across more than one encounter, examples being languages, trends and tastes, and private jokes and stories. They “are symbolic phenomena that have a degree of intersubjective sharing among some (more or less) stable group of individuals” (Sawyer, 2005, p. 216).

Sawyer emphasizes that “the causal power of emergents cannot be explained solely in terms of individual’s representations of them, their demonstrated orientations to them, or their subjective interpretations of them” (Sawyer, 2005, p. 213), adding subsequently that “[a]s levels of reality, stable and ephemeral emergents have an independent, ontological status, and they have causal powers” (Sawyer, 2005, p. 216). Despite having repeatedly emphasized the causal power of emergents, however, Sawyer observes that “the strategic options that the ephemeral frame makes available are limited, and the limiting of the selection set is a form of constraint, although not a strictly deterministic one” (Sawyer, 2005, p. 217). In adding this qualification, Sawyer reinterprets his references to the “causal powers” of emergents in the much more limited terms of top-down constraint, not in terms of deterministic causation as understood in Newtonian mechanics (Sawyer, 2005, pp. 70–2; cf. Arundale, 2020, pp. 217–9).

Although I agree with Sawyer’s argument that drawing on both complex systems theory and computer simulation of artificial societies is one approach among others to studying the emergence of macro-social order in interaction among individuals, conceptualizing the process of social emergence in terms of the mechanism of interactional frames will not prove productive in such research. In considering why that is the case, I turn to another sociologist’s arguments regarding both the place of human interaction in understanding the micro–macro link, and the problematic status of frames in sociological research.

## 3 Rawls on the interaction order, social practices, and conceptual typification

Over three articles Rawls (1987, 1989, 2003) first examines Goffman’s argument that the human interaction order is distinct both from the macro institutional order, and from the micro order of human agency, second critiques Goffman’s frame-based account of interaction, and third provides a basis for an alternative account of the interaction order that draws on Durkheim’s ([1893]1933) recognition of the importance of studying participant’s social practices. Unlike Sawyer, Rawls (1987, 168n9) is not directly concerned with the processes or mechanisms through which emergent properties arise in interaction.

Rawls (1987) argues that “Goffman’s contribution to social theory consists in the idea of an interaction order sui generis which derives its order from constraints imposed by the needs of a presentational self rather than by social structure” (Rawls, 1987, p. 136). More specifically, Goffman argued that the social self is continually achieved in interaction with others, and that the on-going achieving of this presentational self places constraints on the interaction order. Those constraints define the interaction order, “conceived of as a production order wherein a commitment to that order generates meaning. In other words, actions have meaning with respect to the production order rather than in relation to institutionally specifiable ends” (Rawls, 1987, pp. 136–7). Rawls (1987, 146) identifies the interaction order as a social order, *sui generis*, because for Goffman it is “a self-ordered and separate domain, depending upon mutual commitment between actors, which while certainly impinging on macro orders can neither be reduced to, nor entirely explain, aggregate and institutional/structural phenomena.” Rawls (1989) argues that Goffman’s treatment of the interaction order is quite restricted because his “idea of an emergent, constitutive order is worked out around his idea that the self must continually be constituted and reaffirmed in interaction…. This focus on self distinguishes Goffman’s view of the interaction order from the view, held by Garfinkel and Sacks, of a local production order based not on the constitutive achievement of self but, rather, on the constitutive achievement of intelligibility or meaning” (Rawls, 1989, p. 152). Rawls finds both conflicts and confusions across Goffman’s work that she argues can be traced to an understanding of language use in interaction that is “much less original and less interactionally based than his view of self” (Rawls, 1989, p. 153). To address this critique, Rawls turns to Sacks (1992) use of Garfinkel’s (1967) “classical” ethnomethodology (Heritage, 1984; Wilson, 2012; cf. Clayman et al., 2022) in developing an understanding of language use in interacting that is compatible with “the insight concerning a locally produced interaction order and the needs of self that appears in Goffman’s earlier work. Taken together, [Garfinkel’s and Sacks’] work allows for the formulation of a more inclusive and systematic theoretical position with regard to the idea of an interaction order” (Rawls, 1989, p. 153).

In her 2003 article examining constitutive orders of interaction (i.e., orders generated in interacting), Rawls returns to an argument in Durkheim ([1893]1933) that modern social institutions are not organized around the shared beliefs and ritual knowledge of individuals, but are instead organized as groups of persons continually enact distinct sets of situated social practices with one another. One implication of Durkheim’s position is that understanding modern social institutions requires researchers to examine the social practices that persons enact, as opposed to formulating abstractions like beliefs and rituals and attributing these to individuals as the drivers of social behavior (Rawls, 2003, pp. 219–21; cf. Garfinkel, 2007). Rawls finds that because sociologists have not heeded to Durkheim’s argument for attending to situated social practices, the “treatment of practices as ideas, motives, goals, values, beliefs, and the reduction of all those to concepts in the individual mind have become a basic sociological creed” (Rawls, 2003, p. 224), a creed perhaps most clearly represented in Parsons (1937) work, but apparent as well in Goffman’s later work on frames. More specifically, even though Goffman’s (1959) early work did examine some social practices through which individuals accomplished their presentational selves, he “nevertheless continued to ground this process in concepts and typifications to a significant extent” (Rawls, 2003, p. 224). Verhoeven (1985, p. 83) makes the same observation in his examination of Goffman’s work on frames. Rawls (2003, p. 224) continues: “His later attempt to establish a systematic sociology of situations, in *Frame Analysis* (1974) and *Forms of Talk* (1981), became even more conceptual in orientation…. Goffman tended to look only for those details in roles and actions that could be reduced to conceptual types. It is a weakness in Goffman’s position that he tended not to look for social order in the details of practices in their own right” (cf. 232, 234–5, 245–6 and 1987, 146n16, 147).

Goffman is not alone in focusing on “concepts and typifications” rather than on social practices. Collins (1981) points to Garfinkel’s (1967) “radical microsociology” as advancing sociological inquiry by “making it possible to study real-life interaction in second-by-second detail” (Collins, 1981, p. 984), arguing that such study will reveal both “the empirical realities of social structures as patterns of repetitive micro-interaction” (Collins, 1981, p. 985), and that social institutions are only observer’s abstractions that “do not do anything; if they seem to indicate a continuous reality it is because the individuals that make them up repeat their microbehaviors many times, and if the ‘structures’ change it is because the individuals who enact them change their microbehaviors” (Collins, 1981, p. 989, cf. 996). Such observer’s abstractions “can be made fully empirical only by grounding them in a sample of the typical micro-events that make them up” (Collins, 1981, p. 988). Against this background Collins asks what motivates people to repeat such microbehaviors many times, and proposes that they are led to do so by an “underlying emotional dynamics” that “centers of feelings of membership in coalitions” (Collins, 1981, p. 997). These emotions originate in a person’s past participation in chains of interactional rituals—his or her “interaction ritual chains.” More specifically, “[a]n individual who is successfully accepted into an interaction acquires an increment of positive emotional energy. … Acquiring this in one situation, an individual has more emotional resources for successfully negotiating solidarity in the next interaction. Such chains, both positive and negative extend throughout every person’s lifetime” (Collins, 1981, pp. 1001–2). Collins’ account of social structures as patterns of repetitive microbehaviors rests on his identification of interactional ritual chains, emotional energy, and feelings of membership, all of which are abstractions he has formulated and attributed to participants in explaining their behavior. As for Goffman, it is a weakness in Collins’ position that he does not look for social structures in the details of social practices, as both Durkheim and Garfinkel argued.

A clarification is in order here. Rawls is not arguing that concepts have no place in accounting for the interaction order: they cannot be avoided. Instead, Rawls, (2003), p. 224) is arguing that the achieving of action and meaning in interaction “is a process that cannot be accomplished through conceptual typification or theorized accounts. What is required to deal satisfactorily with interaction orders is a notion of practice as concrete and not conceptual.” What Rawls and Garfinkel find problematic are abstract concepts or conceptual types that identify a property that a researcher first formulates so as to gloss the details and contingencies of particular situated activities in order to make the conceptual type widely applicable, and then attributes to participants as the internalized source or driver for their behavior in interaction. For Rawls (2006, p. 6) and Garfinkel it is essential to “see social orders *in* their details as they are achieved in real time *by* persons *through* the enactment *of* those details, instead of through conceptual glosses on these details after the fact.” More specifically, concrete social “[p]ractices are what we can see and hear one another doing. As such, they can be studied directly. Concepts can only be inferred” Rawls, (2003, p. 242). Rawls, (2003, p. 246) adds that “[r]endering practices empirically rather conceptually does not mean that concepts are not used. It means two things: (1) that concepts are not used to *replace* empirically witnessable practices and (2) that social order is not created through the interpretive acts of actors. That is, social actors are not making social order by *using* concepts to *interpret* action.” It follows that like Goffman’s concept of frames, both Sawyer’s (2005) concept of interactional frames, and Collin’s concept of interaction ritual chains are problematic because they treat the achieving of action and meaning, and hence the emergence of social order in everyday social interaction, as accomplished through what I will identify as “conceptual typifications,” rather than as accomplished as participants engage concrete social practices. What is less clear in Rawls (2003) arguments, however, and for the most part in Garfinkel’s (1967), is what comprises these concrete social practices.

Rawls (2003, p. 227) indicates that achieving action and meaning in social interaction requires that participants “construct their social sounds and movements in such a way that they recognizably reproduce courses of practice that are seen by, and expected by, others to mean something particular in the situational context and sequence of events in which they are produced…. What Garfinkel has consistently shown is that this is done through *methods*.” The current understanding of such methods derives largely from Conversation Analysis (CA), as initially developed by Sacks (1992) together with a small group of colleagues and students (Clayman et al., 2022). Conversation analysts have examined a wide range of social practices that include, but are not limited to grammar, phonetics, turn-taking, person reference, membership categorization, nonvocal behavior, sequence organization, overall structural organization, repair, and the relative epistemic, deontic, emotional, and benefactive standings of the participants (Robinson, 2016, p. 6). Conversation analysts have devoted particular attention to four of these domains of practice because they are foundational to all human interaction: practices for turn-taking so that in general only one person talks at a time (e.g., Clayman, 2013; Drew, 2013), practices for forming conversational actions like requesting and granting or asking and responding to questions (e.g., Schegloff, 2007b; Deppermann and Haugh, 2022), practices for repairing problems arising in interaction like mishearings or misunderstandings (e.g., Schegloff, 1987, 1992; Kitzinger, 2013), and practices for the overall structural organization evident in telling a story or in closing a telephone call (e.g., Robinson, 2013). All of these interactional practices are readily observable in talk and conduct, they have been carefully described, and they are repeatedly and reliably employed and recognized by participants across the full range of situations and contingencies they encounter in everyday interacting. These social practices are the methods by which participants both produce and understand talk and conduct in interacting, the methods for production being the same at those for understanding. They comprise the grounds on which participants hold one another accountable/responsible for the actions and meanings that arise in their interacting. And there is now solid cross-language and cross-cultural evidence that the practices of turn-taking (Stivers et al., 2009), action formation (Floyd et al., 2014; Kendrick et al., 2020), and repair (Dingemanse et al., 2015) are universals of human interaction.^4^ As Beach (2022, p. 41) observes, 50 plus years of CA research has revealed “‘the social DNA’ of recorded, transcribed, and translated naturally occurring interactions.”

Sawyer (2005, p. 185) indicates that he employed conversation analytic methods in developing his model of emergence, and he indeed “analyzed conversations” in improvisational theatre (Sawyer, 2003b), but he did not engage CA as exemplified in *The Handbook of Conversation Analysis* (Sidnell and Stivers, 2013) or in CA textbooks. CA is distinct from other methodologies for examining talk and conduct in that the evidence analysts use to ground their understandings of how participants achieve actions and meanings in interacting is exactly the same publically observable evidence that the participants themselves use in understanding one another: the interpretings of prior utterances that they continually display to one another as they place new utterances next adjacent to prior utterances (Arundale, 2020, pp. 223–6; Maynard and Heritage, 2022, p. 19). Conversation analysts use that evidence with the strict admonition to avoid inferences regarding participant interpretings that cannot be directly warranted by the interpretings participants display in their uptake to prior utterances. It is participants who employ social practices in achieving action and meaning in everyday interacting, hence it is their use of practices in achieving their actions and meanings for which analysts need to account. Why not ground those accounts in the very same empirical evidence that the participants themselves employ?

Recall then Rawls’ argument that in developing his account of the presentational self, Goffman identified everyday interaction as an order *sui generis*, distinct from both the micro and macro social orders. Rather than account for the micro order in terms of concrete social practices, however, Goffman adhered to the “basic sociological creed” of accounting for the interaction order using conceptual typifications: “frames” in his case, but “motives, goals, values, beliefs” for other theorists (Rawls, 2003, p. 224). Sawyer (2005, p. 6), seeks an account of emergence that reveals the process and mechanism through which individuals in everyday interaction give rise to macro-level complex systems, but in accounting for that interaction, he too employs a conceptual typification: metapragmatic interactional frames. Collins (1981, pp. 984–5) credits Garfinkel with enabling sociologists to study the specific details of everyday interaction, but in developing his account of the macro order he overlooks Garfinkel and also employs conceptual typification: the emotional energy participants acquire in prior interaction ritual chains (Collins, 1981, pp. 1001–2). Goffman, Sawyer, and Collins are at odds, not only with Durkheim’s ([1893]1933) argument that modern social institutions arise and are maintained as persons continually enact sets of situated social practices with another, but also with Garfinkel’s (1967) position that a satisfactory account of the process of achieving action and meaning in interaction requires examining concrete social practices. Social practices can be studied directly because they are observable, and hence both learnable by observation and instructable (cf. Goodwin, 2018), whereas Sawyer’s interactional frames, Collins’ ritual chains and emotional energies, and other such social cognitive states (Levinson, 2005) that have been posited as intervening between the micro and macro orders are questionably so. Why not avoid introducing conceptual typifications such as these, and instead, following Rawls’ critique, account for the macro-social order as emerging as participants engage observable, concrete social practices in everyday interaction? Section 4 outlines such an account.

## 4 Re-conceptualizing emergence in light of conversation analysis

In view of both Sawyer’s theory of social emergence and Rawl’s critique of accounts based in conceptual typifications, Section 4 offers an account of the emergence of social order in macro-level social systems in terms of the recurrent emergence of social order in micro-level social systems as participants engage observable social practices in everyday talk and conduct. Developing this account involves four steps. In Section 4.1 I examine a transcript of actual talk, first introducing an essential distinction between “operative” and “provisional” interpretings, and then applying that distinction in outlining the Conjoint Co-constituting Model of Communicating: a new model fully grounded in research in CA. In Section 4.2 I use that model in arguing that the social actions and meanings that participants form as they engage in everyday talk and conduct exhibit emergent (non-linear, non-additive) properties that define those participants as a complex social system at the micro-level of two or a few persons. In Section 4.3 I examine why and how the recurrent forming of social actions and meanings in everyday talk and conduct among participants in micro-level social systems, over time and space, offers an account of the formation of emergent properties that define complex social systems at the macro-level of institutions and cultures. In section 4.4 I consider the research needed to further explore this account and to provide empirical evidence.

### 4.1 The conjoint co-constituting model of communicating

Sawyer observes that a “theory of social emergence requires an explicit theorization of symbolic communication and dynamic processes” (Sawyer, 2005, p. 187), but that such a theory is missing both in sociological theorizing on the micro–macro link, and in applying complex system theory in studying it (Sawyer, 2005, pp. 25–6). More specifically, he argues that the accounts of communication employed in computer simulations of social phenomena are too simplistic, or are informed by speech act theory, which he critiques (Sawyer, 2003a). Sawyer does not, however, provide the “explicit theorization” he requires, either of communication, or of the processes involved in his account of an actor’s use of implicit metapragmatic strategies to create frames that have causal effects on subsequent interaction (cf. Arundale, 2020, pp. 189–90). Research in CA provides not only the conceptual framework, but also the empirical grounding for an explicit theorization of human communicating in everyday interacting that takes the form of a sequential/procedural model specifying the process and mechanism of the emergence, in interacting among two or a few participants, of properties that define those participants as a complex system.

**Figure 1:**
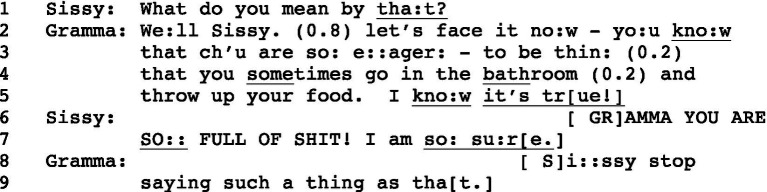
SDCL: Gramma/Sissy (Beach, 1996, p. 116).

Outlining that model of human communicating in this section involves examining an excerpt from everyday interacting, and in doing so introducing two concepts that are essential to tracing in detail how the participants engage social practices to conjointly co-constitute action and meaning in a particular sequence of talk. In Figure 1, a university-aged granddaughter (Sissy) is talking with her grandmother (Gramma), who is a nurse. In his book length analysis of this 13-min. conversation, Beach (1996) argues that over the two and a half minutes that precede Figure 1, Sissy becomes aware that their conversation centers around a problem in her behavior regarding food, although Gramma does not explicitly identify that behavior, and Sissy does not explicitly deny having the problem. The conversation begins with talk about Sissy’s work hours and exercise needs, then shifts to comments by Gramma about Sissy’s thinness and weight loss. This leads to a discussion of Sissy’s eating habits at a recent meal they shared, and of her appearance in preparation for her upcoming wedding. Sissy states that she is not going to lose any more weight and assures Gramma that “I’ll eat just fine.” Gramma agrees that Sissy always eats well, but asks “What happens to the food that you eat?” and adds that “You’re not getting any bigger.” Sissy then poses the question in line 1 of Figure 1. Both women overlap one another (marked by vertically aligned brackets) and stretch out certain sounds (marked by “:”), and Gramma pauses briefly in line 2 (0.8 s). Returning repeatedly this excerpt will prove essential in following the discussion.^5^

As its speaker, Sissy creates an interpreting of her first position utterance in line 1 as she designs and produces it for Gramma. As recipient of her first position utterance, Gramma also creates an interpreting in listening to it. Sissy and Gramma are cognitively (and emotionally) autonomous from one another, just as are all participants in interaction, hence they have no direct access to one another’s interpreting of any utterance. Like all utterances and visible conduct in interacting, Sissy’s first position utterance enables a small range of potential interpretings, but does not limit those interpretings to a single definitive understanding. There is always some openness in how an utterance might be interpreted, as for example in the different possible referents of the word “that.” At the moment she is designing and producing her first position utterance, Sissy has no knowledge regarding the particular interpreting Gramma is constructing and can only presume that Gramma will interpret her utterance as she has designed it to be interpreted. As she listens, Gramma likewise has no knowledge regarding Sissy’s particular interpreting of her first position utterance and can only presume that Sissy interprets it as she does. Evidence regarding how another person has interpreted one’s utterance becomes available only in the subsequent unfolding of the particular sequence of utterances they co-constitute.

There is a long tradition of conceptualizing Sissy’s and Gramma’s interpretings of Sissy’s first position utterance solely as cognitive phenomena that arise in their individual processing of any given utterance. The Conjoint Co-constituting Model of Communicating or CCMC (Arundale, 2020, Chap. 3) outlined in this section breaks from that tradition in understanding their interpretings not only as cognitive phenomena, but also as social phenomena that arise in interacting with one another. From this distinct perspective, it is apparent both that each participant’s initial cognitive interpreting of a given utterance in interacting is always a *provisional* interpreting, and that it remains provisional until that participant has gained some knowledge of the particular interpreting accorded to that utterance by another participant. Apart from such knowledge, an initial interpreting is either a speaker’s projection of the interpreting a recipient will create for the speaker’s utterance, or a recipient’s initial interpreting of a speaker’s utterance. A provisional interpreting becomes an *operative* interpreting at the point a speaker gains knowledge regarding how his or her own utterance has been interpreted by a recipient, or a recipient gains knowledge about how the speaker had interpreted his or her own utterance. An operative interpreting is one that is functional or useable for carrying on in an evolving sequence of utterances because it takes into account the interpreting that another participant has evidently accorded to it. At a later point in the same conversation, or in a different conversation, the operative interpreting of any specific utterance may well be different in view of subsequent evidence regarding the interpreting accorded to it.

The distinction between provisional and operative interpretings is original to the CCMC, and essential in enabling one to trace in detail the moment-by-moment, sequential evolution of each of the participant’s interpretings of the action and meaning of a given utterance, as the participants place each new utterance next adjacent to the prior utterance in a sequence of utterances. In other words, distinguishing between provisional and operative interpretings enables one to examine in detail the procedural development of Gramma’s and of Sissy’s interpretings of each utterance, both as a cognitive process and as a social process, as they alternate in adding each new next adjacent utterance. It is the CCMC’s account of the procedural development of participant’s operative interpretings that provides the basis for the procedural account offered in this chapter of the emergence of social order.

**Figure 2:**
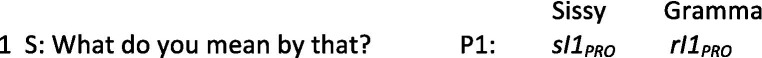
One first position utterance.

As participants engage one another in interacting, they constitute the shape and sequence of their turns, the conversational actions their utterances are taken as accomplishing, and what those utterances are taken to mean, all at the same time. It will simplify things to focus on Sissy and Gramma’s mutual constituting of just the action and the meaning of Sissy’s first position utterance in line 1 in Figure 1. Sissy designs her first position “What do you mean by that?” as a *wh*-question that implements the social practice of requesting and granting/denying, from among the broader set of practices for recruiting assistance (Kendrick and Drew, 2016). In designing the first pair part of an adjacency pair as a potential request, she projects that Gramma will provide a granting as the second pair part in which Gramma makes explicit what she had meant in asking “What happens to the food you eat?” followed by “You’re not getting any bigger.” Sissy’s interpreting of her own utterance is at this moment provisional because while she may be quite sure she is requesting an explication, she as yet has no knowledge of how Gramma will understand the utterance. Gramma’s interpreting of Sissy’s utterance, as Sissy vocalizes it, is likewise provisional because she as yet has no knowledge of how Sissy has interpreted her own utterance. Figure 2 presents both women’s interpretings of this first position utterance (P1) in schematic form: “*sI1_PRO_*” represents Sissy’s provisional interpreting of utterance 1, where “*s*” denotes the utterance’s speaker, “*I1*” denotes her interpreting of the first position utterance, and both the subscript “*_PRO_*” and italics identify that interpreting as provisional. Similarly, “*rI1_PRO_*” represents Gramma’s provisional interpreting of the first position utterance as its recipient, denoted as “*r*.”

**Figure 3:**
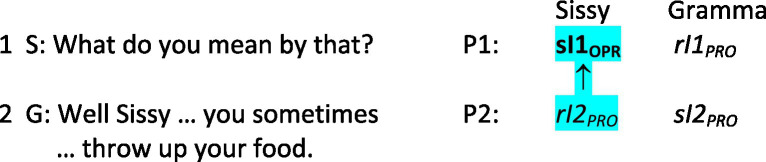
Two next adjacent utterances.

Gramma designs her next adjacent second position uptake in lines 2 to 5 of Figure 1 by drawing on the same social practice of requesting and granting/denying that Sissy utilized for utterance 1, projecting that in being very explicit about what she had meant, Sissy will understand her as granting the potential request. Gramma’s opening “Well” draws on the practices of well-prefacing of utterances (Schegloff and Lerner, 2009), in this case alerting Sissy that this second position uptake to the request requires Sissy’s special attention. Gramma then attributes to Sissy knowledge both of her own motivation for and of her own behavior in throwing up her food, and adds an assertion that this attribution is true. Together these projections for interpretings of action and of meaning comprise Gramma’s provisional speaker interpreting of her own second position uptake, represented schematically in Figure 3 as Gramma’s “*sI2_PRO_*.”

Gramma’s second position utterance is central in conjointly co-constituting action and meaning because it provides Sissy with evidence of how Gramma has interpreted Sissy’s first position utterance. As its recipient, Sissy gauges whether Gamma’s second position utterance falls within the range of relevant next actions in view of the social practice of requesting and granting/denying. In this case it does, and it provides Sissy with confirmation that Gramma has taken the first position utterance as an action of “doing requesting,” and more specifically to have been a request to be explicit about what Gramma had meant: Gramma perceives her as being bulimic. As Gramma completes the second position utterance, Sissy’s prior provisional interpreting of her first position utterance becomes an operative interpreting because she now has evidence of how Gramma has interpreted it at this point, within the specific circumstances of their conversation. This newly formed operative interpreting is represented schematically in Figure 3 as Sissy’s “**sI1**_**OPR**_” (highlighted in blue) where both the subscript “_**OPR**_” and bold face designate it as an operative interpreting, and where the vertical arrow below it (↑) indicates that her newly formed operative interpreting arises directly from and is dependent upon her interpreting of Gramma’s second position utterance (i.e., Sissy’s “*rI2_PRO_*”). Note very importantly that at this point Gramma’s interpreting of Sissy’s first position utterance remains provisional because Gramma as yet has no evidence of how Sissy had interpreted her first position utterance.

**Figure 4:**
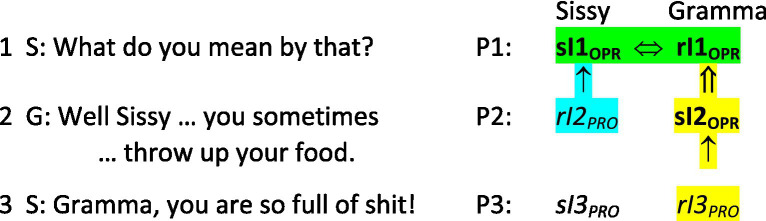
Three next adjacent utterances.

Sissy designs her next adjacent third position utterance, “Gramma you are so full of shit! I am so sure” (lines 6–7) projecting that Gramma will interpret it as a next action relevant to Gramma’s second position utterance. Following Beach’s (1996) analysis, Sissy’s third position utterance is an outright discounting of what Gramma has just attributed to her, that discounting accomplished first by drawing on the social practices of denigrating others, in this case by characterizing Gramma as “full of shit,” and second by insisting that that is the case. By implication, Gramma’s attribution has no viable basis, although Sissy has stopped short of directly denying what Gramma has alleged. Sissy’s third position utterance is equally central in conjoint co-constituting in that it provides Gramma with evidence of how Sissy has interpreted Gramma’s second position utterance. Gramma now draws on the same social practices of denigrating to gauge whether Sissy’s third position utterance is a responsive next action. In this case it is, and it provides Gramma with clear evidence that Sissy has interpreted Gramma’s second position granting as an explicit attribution that she is bulimic, and that Sissy utterly rejects that attribution. At this point Gramma’s provisional interpreting of her own second position uptake becomes operative in that she now knows how Sissy has taken it. As in Figure 4, Gramma’s newly formed operative interpreting is denoted as “**sI2**_**OPR**_” (highlighted in yellow), and it is dependent upon Gramma’s interpreting of Sissy’s third position utterance (Gramma’s “*rI3_PRO_*,” also highlighted in yellow).

But Figure 4 indicates that much more is happening as Sissy places her third position utterance next adjacent to Gramma’s second position utterance. Because Gramma now knows that Sissy has interpreted Gramma’s second position utterance as granting Sissy’s request to be explicit, Gramma also has confirmation that Sissy’s first position utterance was indeed a question requesting an explication of meaning. As in Figure 4, Gramma’s newly formed operative interpreting of her second position utterance (her “**sI2**_**OPR**_”) enables her to form an operative interpreting of Sissy’s first position utterance (her “**rI1**_**OPR**_”), this latter interpreting having remained provisional until this point. The double vertical arrow (**⇑**) below this newly formed operative interpreting of Sissy’s first position utterance denotes that it arises as Gramma makes an inference based on her newly formed **sI2**_**OPR**_, which in turn is directly dependent on upon her *rI3_PRO_*, which she has just now formed in interpreting of Sissy’s third position utterance (Arundale, 2020, pp. 80–2).

At the point Sissy’s third position utterance is complete, then, both Sissy and Gramma have formed operative interpretings of Sissy’s first position utterance, the double-headed arrow (**⇔**) between Sissy’s “**sI1**_**OPR**_” and Gramma’s “**rI1**_**OPR**_” (highlighted in green) denoting that their respective interpretings of Sissy’s first position utterance are interdependent (i.e., reciprocally dependent) because both women’s operative interpretings of the first position utterance derive from or are conditional upon their interpretings of the *same two next adjacent utterances*. Following the understanding of human communicating employed here (Arundale, 2020, Part 1), Gramma and Sissy have *conjointly co-constituted* their respective interpretings of the conversational action and meaning of Sissy’s first position utterance “What do you mean by that?” Note the asymmetry involved in this triad of next adjacent utterances: As *speaker* of the first position utterance, Sissy needs only Gramma’s second position utterance to provide the evidence needed for her to confirm (or modify) her provisional interpreting of her first position utterance so that it becomes operative. But as *recipient* of Sissy’s first position utterance, Gramma must await Sissy’s third position utterance to obtain the evidence she needs to confirm (or modify) her provisional interpretings not only of her own second position utterance, but also in turn of Sissy’s first position utterance.

In the Conjoint Co-constituting Model of Communicating, *“communicating” is the process through which both the speaker and the recipient(s) create operative interpretings of a given first position utterance, those operative interpretings arising only at the point the participants have designed and delivered two further next adjacent utterances in a triadic sequence.* The CCMC directly reflects Garfinkel’s early recognition of the importance of third position utterances in human communication, as in Rawls (2006, pp. 29–33, 184), Arundale (2020, pp. 89–93), and Heritage (2018, pp. 30–1). As used here, the term “co-constituting” refers to the unique processes engaged when one individual forms perceptions and interpretations of the activities of another human being: processes that are not engaged for non-human entities (Arundale, 2020, pp. 53–54; 409–12). The term “conjoint” points to the non-linear, non-additive, sequential entwining of two or a few individual’s processes of co-constituting in interacting (Arundale, 2020, pp. 53–6), as distinct from additive “joint” activity.

**Figure 5:**
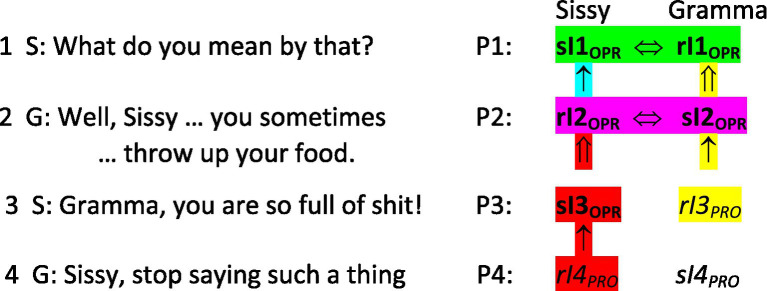
Four next adjacent utterances.

Looking beyond this first triad of utterances, as in Figure 5, Gramma’s fourth position reprimand of Sissy (lines 8–9 in Figure 1) completes a new, overlapping triad of next adjacent utterances that provides the evidence Sissy needs to create an operative interpreting of her own third position utterance (her “**sI3**_**OPR**_,” highlighted in red). That operative interpreting in turn provides the Sissy with the basis for forming an operative interpreting of Gramma’s second position utterance (Sissy’s “**rI2**_**OPR**_”), at which point Sissy and Gramma have conjointly co-constituted their respective operative interpretings (highlighted in magenta) of the conversational action and meaning of Gramma’s second position utterance across this second, overlapping triad of next adjacent utterances. Both women now have evidence that Gramma’s second position utterance identifies Sissy as bulimic.

I examine this conversation in more detail, particularly with regard to its implications for Gramma and Sissy’s relationship, in Arundale (2020, pp. 8–12, 170–76, 190–6, 339–48). Gramma and Sissy do achieve some degree of overlap in interpreting with regard to conversational action, but same operative interpretings for a given utterance are not a necessary outcome of conjoint co-constituting (Arundale, 2020, pp. 96–102). Both complementarity and difference in participant operative interpretings are also normal, everyday outcomes of conjoint co-constituting. For example, although both women interpret Gramma’s second position utterance as identifying Sissy as bulimic, they differ markedly with regard to whether that attribution is correct. The processes of conjoint co-constituting provide participants with evidence of how their own utterances are being interpreted, and of how another’s utterances are to be interpreted. That evidence may lead the participants to bring different interpretings into overlap, to recognize that their interpretings remain distinct, or to assume overlap when there is difference, or difference when there is overlap. As a model of human communicating, the CCMC is a substantial departure from commonly held understandings of communication in terms of the transmission of information or of the encoding and decoding of meanings, both of these models presuming that the outcome of communication is identity between speaker and recipient meanings (Arundale, 2020, pp. 237–8).

In the most general terms, then, the Conjoint Co-constituting Model of Communicating offers an account of how participants conjointly co-constitute operative interpretings of any given utterance over triads of utterances in next adjacent positions, those triads successively overlapping prior triads of utterances as each new next adjacent utterance is added in sequence. Conjointly co-constituting operative interpretings provides a speaker with evidence of how a recipient has interpreted the speaker’s first position utterance, and a recipient with evidence of how a speaker had projected the first position utterance would be interpreted. Operative interpretings are central to the progressivity of everyday interacting when a recipient’s operative interpreting corresponds with a speaker’s projection, but that centrality is even more apparent when a recipient’s operative interpreting departs from a speaker’s projection, as in Arundale (2020, pp. 79–88). I examine the CCMC in depth, and its grounding in research in CA, in Part 1 of *Communicating & Relating* (Arundale, 2020, 2021), with a visual representation in Appendix 2. Six further observations about the model are important before examining it with regard to micro-level emergence in section 4.2.

First, the terms “participant,” “utterance” and “position” have specific definitions as they are employed in the CCMC. A *participant* is a person who engages both in interpreting another’s utterances in sequential interacting, and in designing and delivering utterances for another person to interpret. As he or she delivers an utterance for another person to interpret, and therein becomes accountable/responsible for the interpretings of that utterance, a participant becomes an *agent*, and exhibits his or her *agency* (Arundale, 2020, p. 187). All accounts of human communication are formulated by *observers*, but following the practice in research in CA, the CCMC is an observer account formulated from the perspective of the participants/agents engaged in everyday interacting (Arundale, 2020, pp. 223–6).

Second, although an “utterance” is often understood as a turn at talk, or perhaps as a turn constructional unit, Schegloff (2007b, p. 15) points to other elements of talk like words, syllables, and sounds at a finer level of granularity in the sequential organization of interacting. Research has extended this list to include not only elements such as aspirations, laughter, false starts, silent receipts, and continuers like “uh huh,” but also and very importantly, the whole range of nonvocal elements of gesture, gaze, and bodily movement and position. These nonvocal elements may stand apart as distinct elements in a sequence, or may co-occur with vocal elements without interfering with them. Both prior and current research make clear that all of these elements are fully consequential in everyday interacting (cf. Arundale, 2020, pp. 143–53, 330–9; e.g., Deppermann and Streeck, 2018; Goodwin, 2018). In short, an element like an aspiration, a silent receipt, a headshake, or a hand movement may well be an utterance occupying a position in a sequence of interaction. Within the CCMC, then, an *utterance* is defined as *a vocal or nonvocal activity by one participant in sequential interacting, or the occasioned absence of such an activity, that may but need not overlap another participant’s activity* (Arundale, 2020, pp. 50–1).

Third, a “position” in interaction is the location in a sequence at which an element appears, but what comprises a position depends on the term’s use with regard to a particular normative order of interaction such as turn-taking, action formation, or repair. With regard to turn-taking, next adjacent turns are in next adjacent positions; with regard to action formation, the first and second pair parts are often in next adjacent positions, but those positions may become separated by intervening utterances; and with regard to repair, the four-position “repair initiation opportunity space” (Schegloff, 1992) identifies the locations at which a participant might initiate repair on a problematic aspect he or she identifies in a given first position utterance. Within the CCMC, then, a *position* in a triad of utterances is defined as *(a) an utterance, as above, (b) that is recognizable and in most cases interpretable in terms of a normative order of organization, and (c) organized as any given first utterance and the two next adjacent utterances following it, (d) where any two next adjacent utterances of the three utterances are produced by different participants.* In Gramma and Sissy’s interacting, the utterances comprising the three-position triads correspond with three next adjacent turns at talk, but that need not be the case if one participant produces two or more successive turns, or produces a headshake or a nod in overlap with another participant’s verbalization (e.g., Arundale, 2020, pp. 143–53, 330–9).

Fourth, as outlined above, the CCMC describes the time-ordered process by which two or a few participants establish their respective interpretings of action and meaning, or in other words, their respective senses of the state of the talk as each new utterance appears in sequence. The organizing framework basic to triadic conjoint co-constituting is the fundamental “default principle” of nextness, adjacency, and progressivity in sequential interacting: the principle that each element added to a sequence “should come next after the prior,” and be “hearable [and/or seeable] as a/the next one due” (Schegloff, 2007b, pp. 14–5; cf. Arundale, 2020, pp. 48–50). As Schegloff (2007b, p. 15) elaborates, “[s]hould something intervene between some element and what is hearable as a/the next one due … it will heard as qualifying the progressivity of talk and will be examined … to find how it reaffirms the understanding-so-far of what has preceded, or favors one or more of several such understandings that are being entertained, or how it requires reconfiguration of that understanding.” Unlike the normative organizations of turn-taking, action sequencing, and repair initiation, the organizing principle of adjacency, nextness, and progressivity is always in play as each new element, at whatever level of granularity, is added next adjacent to a prior element in an evolving sequence of elements. It follows that triadic conjoint co-constituting is continually occurring as new utterances appear in next adjacent positions (Arundale, 2020, pp. 52–3, 72–86). In other words, as long as two or more participants continue to provide uptake to one another’s utterances, there is “no time out” from communicating.^6^

Fifth, one implication of Schegloff’s default principle of nextness, adjacency, and progressivity is that at all levels of granularity, sequences in human interacting are designed and interpreted on a moment-by-moment basis as participants place utterances next adjacent to the utterances of other participants. One’s designing of a new utterance to be placed in sequence next adjacent to another’s prior utterance involves *projecting* how that new utterance is likely to be interpreted in relation to the prior utterance (Deppermann and Streeck, 2018, p. 6), and one’s interpreting of a new utterance placed next adjacent to a prior utterance involves *assessing* how that new utterance is related to that prior utterance. Projecting and assessing happen in the moment of interacting, as each new element is added, the final form of any added element being unknown until that element is complete. In projecting and assessing the “nextness” of each new adjacently placed utterance, participants draw directly on the concrete social practices for interacting that they presume they share. At any moment multiple practices may be play, and if so they may or may not be consistent with one another. A core set of these practices are universals of interaction, as in Section 3, with others having the status of normative social practices in cultural or language groups that allow persons to interact productively with persons in the group they have never before encountered (Arundale, 2020, p. 49). Participants like Gramma and Sissy draw on their knowledge of social practices in incrementally ordering their particular sequence of interaction, each person’s new, next adjacent utterance moving the sequence along, and providing the bases both for conjointly co-constituting operative interpretings of prior utterances, as well as for designing subsequent utterances. Those operative interpretings often reaffirm interpretings-so-far, but as Schegloff (2007b, p. 15) notes, they may just as well refine, redirect, or reconfigure those interpretings, or at times terminate the interpreting-so-far.^7^ Very importantly, even though Gramma and Sissy both directly affect the unfolding of their conjoint co-constituting, neither of them controls that unfolding because the incremental ordering of their sequence of interaction could have taken a very different direction had one of them provided a different next adjacent utterance at any point.

Sixth, considering the participant’s use of social practices in the moment-by-moment incremental ordering of a sequence serves to clarify what Rawls (2003, p. 227, cf. Krippendorff, 1970) contends in noting that “[f]rom Garfinkel’s perspective, interactional practices do not *constrain* action, or practice, in any case. They *order* it, make it recognizable and thereby intelligible.” Taking the organization of conversational action (Schegloff, 2007b) as a case in point, a first participant who designs what he or she provisionally interprets as a request draws on the social practice of requesting and granting/denying to project that the next adjacent utterance of the addressed participant will be a granting or a denying of that request. Participants can reliably anticipate that others in their community of language users know this social practice, among the many others across the domains noted in Section 3, hence it might appear that the social practice constrains the addressed participant to constructing that next adjacent utterance as a granting or a denying. But understood in terms of the CCMC, a first participant’s drawing on this social practice in designing an utterance does no more than enable his or her projection of the next adjacent utterance as a grant or denial. There is nothing in the first participant’s provisional interpreting or in the composition of his or her utterance that determines how the action-in-progress will eventually be realized. It is entirely possible that as the first participant draws on his or her provisional projection in assessing whether or not the addressed participant’s next adjacent utterance is a grant or denial, he or she will find the interpreting-so-far reconfigured as something other than a request, or perhaps find that action terminated altogether. In Figure 1, Sissy’s first position *wh-*question potentially implements the social practice of requesting followed by granting/denying, but in second position Gramma could well have responded to the *wh*-question in a manner that continued her practice of not explicitly identifying what she sees as Sissy’s bulimia, just as she had done over the prior 2 min. of their conversation (cf. Fox and Thompson, 2010). Were Gramma to have done so, Sissy’s operative interpreting of her own first position utterance would be as a *wh*-question, not as a request, and would provide a very different basis for designing her next adjacent third position utterance.

It is in this sense that social practices *order* or open possibilities for sequences of conversational action in interacting. A participant’s projection does not *constrain* or determine the type of action implemented by the next adjacent utterance because the projected action can be realized, modified, or obviated only in view of the actual utterance another participant provides in the next adjacent position (cf. Arundale, 2020, pp. 217–9). The principle applies not just to action formation, but to all domains of social practice, including that of overall structural organization (Robinson, 2013), which might appear to involve some type of script, ritual, frame, or similar sequential formula understood account for overall sequences of utterances in interacting. As in Section 3 with regard to “frames,” such sequential formulae are an observers’ conceptual typification of a sequence of interactional practices that participants are presumed to follow in a lock step manner upon identifying the type of situation in which they are involved. Participants in everyday interacting do not passively follow such sequential formulae, but instead engage actively in conjointly co-constituting every sequence of utterances anew, moment-by-moment, in light of the contingencies that pertain to that specific situation. That is the case even if they are once again co-constituting the “same” overall sequence they have constituted before (Arundale, 2020, pp. 231–3).

### 4.2 The emergence of micro-level order in everyday interacting

The term “emergence” is most commonly used in the sense of a phenomenon coming into being over time in some activity. In this sense, the Conjoint Co-constituting Model of Communicating is an account of the emergence, across triads of next adjacent utterances, of two or a few participant’s operative interpretings of any given first position utterance (Arundale, 2020, pp. 85–6). Yet “emergence” is also used, and will be used herein, in the additional, technical sense of the coming into being over time of properties that define complex social systems. In this Section I argue that *the CCMC’s account of the emergence of participant operative interpretings across three position triads is also an account, in the additional, technical sense, of the emergence of key, non-linear, non-additive properties that define two or a few participants as a micro-level, complex social system.* This account of emergence at the micro-social level provides the basis for the account in Section 4.3 of the emergence of social order at the macro-social level. Examining emergence in interacting at the micro-level involves two steps. First, I consider four emergent properties of complex systems in general that distinguish them from additive collectivities, and indicate how each property is apparent in Gramma and Sissy’s conjoint co-constituting of operative interpretings, thereby defining them as a micro-level, complex social system. Second, I identify four additional, more specific emergent properties of everyday interacting likewise apparent as participants form operative interpretings across triads of next adjacent utterances. I draw on Sawyer’s (2005, pp. 94–7) discussion of the four general properties as representative of many similar overviews (e.g., Clark, 1997, pp. 103–13): non-aggregativity, non-decomposibility, non-localizabilty, and complexity.

*Non-aggregativity* in a complex system refers to the inability to substitute the parts of a system for one another, to add or remove parts from the system, or to rearrange the parts without changing the system’s overall properties, as well as the presence of facilitative or inhibitory interactions among a system’s parts such that a change in one part affects the system as a whole. In the social systems that emerge as two or a few participants place utterances next adjacent to those of other participants, the “parts” of the system are the individual participants and the particular utterances each designs and delivers for others to interpret, these person/utterance parts being directly observable. In Figure 1, Gramma is one unique person/utterance part and Sissy is the other, neither of which can be substituted for the other. Removing one of them from the dyad would obviously destroy the dyadic system, but just as clearly adding a new person/utterance part to a dyad, or removing one person/utterance part from a conversation among three or more participants, would change the operative interpretings that would evolve as each next adjacent utterance is added. Rearranging the sequencing would have a similar effect. Examining the evolution of Gramma and Sissy’s provisional and operative interpretings of action and meaning across successive triads of utterances makes apparent that each person/utterance part confirms (facilitates) or alters (inhibits) the other part’s interpreting of prior utterances. Non-aggregativity is one characteristic of two or a few participant’s operative interpretings of the action and meaning of any given first position utterance that defines the participants as a micro-level, complex social system.

*Non-decomposability* of a complex system is present where the “overall system organization is a significant influence on the function of any component” (Sawyer, 2005, p. 96), where the parts of the system are interdependent, or where the behaviors or states of one part are reciprocally conditional on the behaviors or states of other parts. Returning to Gramma’s and Sissy’s conversation and the evolution of their respective operative interpretings of Sissy’s first position “What do you mean by that?” (line 1 in Figure 1), Sissy’s operative interpreting is conditional on Gramma’s second position uptake, whereas Gramma’s operative interpretings, not only of her own second position uptake, but also of Sissy’s first position utterance, are conditional on Sissy’s third position uptake. At the point the first triad of utterances is complete, Gramma’s and Sissy’s operative interpretings are not only mutually or unilaterally conditional on one another’s subsequent utterances, but also and more specifically, they are reciprocally conditional on the same set of subsequent utterances (Arundale, 2020, pp. 78–84). The triadic sequential organization of Figure 1 is both the central factor in forming the interdependency of Gramma and Sissy’s respective operative interpretings of action and meaning for the first position utterance, and an example of the spontaneous self-organization in complex social systems that generates both order and interdependency. Krippendorff (1984, p. 29; 2009, p. 43, cf. Arundale, 2020, pp. 29–32) defines “communication” as “that observer-defined relational construction which explains what makes a system defy its decomposition (without loss of understanding) into independent parts.” Non-decomposability is a second characteristic of two or a few participant’s operative interpretings of action and meaning that defines the participants as a micro-level, complex social system.

*Non-localizability* in a complex system in present where there are properties of the system that cannot be identified with or localizable within particular parts of the system. Clearly Gramma’s and Sissy’s interpretings, whether provisional or operative, are their own cognitive/emotional property as individual persons. However, unlike their provisional interpretings, their operative interpretings of action and meaning for any given utterance are also properties that are not localizable solely within the individual persons involved because those interpretings evolve only as they interpret the utterance the other person places next adjacent to that given utterance. A different next adjacent utterance would lead to a different operative interpreting. Non-localizability is a third characteristic of two or a few participant’s operative interpretings of action and meaning that defines the participants as a micro-level, complex social system.

*Complexity* is apparent where the rules of interacting among the parts are multiple and complicated, one key index of complexity being the non-linearity in the processes of interacting that is evident, for example, where the outcomes of those processes are not predictable from the initial states of the process, or where different outcomes result from essentially the same initial states (cf. Sawyer, 2005, p. 97; Clark, 1997, p. 236). Clearly the “rules” of human languages and of the social practices of engaging them in interacting are multiple and complicated. Schegloff (1981, p. 89) argues that any sequence of utterances the participants actually create is one among a number of “contingent alternatives” they could have created, making it essential for analysts to retain “a sense of the actual as an achievement from among possibilities.” As noted above, the sequence of operative interpretings of action and meaning that Gramma and Sissy conjointly co-constitute could have evolved in many different directions following line 1 in Figure 1 had either of them delivered a different next adjacent utterance at any position (Heritage, 1984, p. 263). Their sequence is not predictable because it is the outcome of the non-linear process of conjointly co-constituting operative interpretings. Again, starting from ostensibly the same initial utterance, “What do you mean by that?” designed in view of a widely recognized social practice for making requests, provides no guarantee that the next adjacent utterance will be a grant or a denial. Complexity, understood as non-linearity in the processes of interacting, is a fourth characteristic of participant’s operative interpretings of the action and meaning of a given utterance that defines two or a few participants in everyday interacting as a micro-level, complex social system.

Examining how these four generic emergent properties of complex systems are evident in everyday interacting among two or a few participants makes apparent that *the participant’s operative interpretings of the action and meaning of any given first position utterance that evolve across triads of next adjacent utterances are the central emergent property of everyday interacting that defines those participants as a complex system*. Yet beyond these four abstract properties of all complex systems are number of other emergent properties specific to the micro-level systems that participants form as they engage in everyday interacting. Brief descriptions of four such properties must suffice, as detailed examinations lie well beyond the scope of this chapter:

1. Concomitant with emergent operative interpretings of action and meaning in micro-level systems are emergent *operative interpretings of relationship,* or more precisely of “relating,” as an on-going, dynamic process of both connecting with and separating from one another, separating being the dominant pole for Gramma and Sissy in Figure 1 (Arundale, 2020, Chaps. 7–9).
2. The emergent creating, sustaining, and changing of *individuality*, or in other words, the emergence in interacting with other persons of the complex systems that are individual human vis-a-vis other human selves (e.g., Arundale, 2020, pp. 202–6; Rawls, 2006, pp. 21–4, 110–4). Eleven utterances beyond Sissy’s third position denial in Figure 1, Gramma and Sissy conjointly co-constitute Sissy’s highly qualified admission that she is bulimic (Arundale, 2020, pp. 339–48).
3. The emergent *sequential ordering of utterances in interacting*, as examined in Section 3 (Rawls, 2003, p. 227), or in other words, the emergent progressivity of talk and conduct (Schegloff, 2007b, p. 15) in everyday interacting.
4. The emergent *commonality* in social practices and in meanings among participants in micro-level (and macro-level) complex systems (Arundale, 2020, pp. 176–82). This property is important in Section 4.3 and warrants further consideration.

What I identify as “commonality” in social practices and in meanings is fundamental in enabling participants, as they design or interpret utterances in interacting, to reliably assume that other participants know the social practices and meanings regularly employed in their community of language users, whether small or large. More specifically, commonality is not what is generally known as common ground or mutual knowledge, nor is it some type of core or literal meaning, nor is it “intersubjectivity” in the sense of “treatably same interpretings” (Arundale, 2020, pp. 95–102). Commonality in social practices and in meanings is an emergent property of everyday interacting that arises over time among participants as they recurrently engage social practices in conjointly co-constituting operative interpretings of action and meaning. Gramma and Sissy’s conversation reveals that they have some degree of commonality in their meanings for persons who are typical of those with bulimia (Beach, 1996, p. 46), but because they acquired their respective meanings in quite different communities of language users, the extent of overlap in their meanings is likely very limited. Their conversation also reveals a high degree of commonality in their understandings of the social practices involved in formulating and granting/denying requests. Participants routinely presume commonality in social practices and in meanings as they design and interpret utterances in everyday interacting, but its presence or absence can be established only as those participants conjointly co-constitute interpretings of a given utterance at a given moment in interacting (Arundale, 2020, pp. 176–82). If commonality is not present the participants will likely engage the practices of repair, and the operative interpretings that the participants form in the course of doing so may well be instrumental in establishing commonality for subsequent interpreting. Deppermann and Schmidt (2021) use CA in examining the evolution over 20 theatre rehearsals of what I identify as “commonality” in meaning among a small group of actors for the Japanese esthetic concept *wabi sabi*, beginning with the director’s initial introduction of this previously unknown term. Deppermann and Schmidt employ the term “common ground,” but provide an extended critique of that concept and eventually adopt the term “commonality” in its place.

Each of the emergent properties sketched above originates and is organized in the interacting among two or a few participants, defining them as a complex, micro-level social system. None of these properties belongs to or is defined solely upon the participants as individuals. Each property is a different facet of *the emergence of micro-level social order in everyday interacting.* Because this micro-level social order defines two or a few participants as a complex system, and because that micro-level order emerges *only* as those participants interact with one another, it follows that when the participant’s interacting terminates, their system *qua* system ceases to exist. Provided however that the participants have established commonality in the social practices and meanings they have engaged in past interacting, they can re-create and thus sustain that system by resuming interacting and re-engaging the same social practices and meanings. Social systems of two or a few participants are therefore episodic, and sustained only in recurrent episodes of interacting among the participants. Absent a lens suitable for looking for it, we have not noticed micro-level social order continually emerging around us in everyday face-to-face interacting. Sawyer (2005) and Collins (1981) both look at face-to-face interacting in their search for accounts of macro-level emergence, but their lenses are not focused on the observable social practices that enable participants to interact every day.

### 4.3 The emergence of macro-level social order in light of conversation analysis

Building directly on the above account of the emergence in everyday interacting of a range of properties that define micro-level complex social systems of two or a few individuals, I argue in this section that *the CCMC’s micro-level account of emergence provides the basis for an account of the emergence, in recurrent talk and conduct over time and space, of properties that define the macro-level social systems that are social institutions and cultural groups.* Again, the basic idea is simple: a micro-level complex system emerges as two or a few individuals conjointly co-constitute actions and meanings in an episode of interacting at some particular time and place, and a macro-level complex system emerges as a larger number of individuals recurrently conjointly co-constitute actions and meanings across multiple episodes of interacting occurring over time and space. Also again, the basic idea is not new: Collins (1981, p. 985), for example, draws on Garfinkel in arguing that social institutions rest on “patterns of repetitive micro-interaction.” What is new in the re-conceptualization offered in this Section is accounting for the emergence of macro-level social order in terms of the recurrent emergence of micro-level social order as participants engage observable social practices.

More specifically, a macro-level social system is created, sustained, and changed as persons in a larger community recurrently engage social practices and meanings associated with that macro-level social system, and for which they have previously established commonality, across multiple episodes of micro-level interacting distributed over time and over space, the scope of that commonality establishing the scope of the macro-level social system (Arundale, 2020, p. 177). Like micro-level systems, then, macro-level systems are episodic in that for the participants in a given micro-level system, the macro-level social system *qua* system ceases to exist when their interacting terminates, or when they cease engaging the social practices and meanings associated with that macro-level system. Presuming they have established commonality in past interacting in the social practices and meanings associated with the macro-level system, however, the participants in a given micro-level system can, at any particular time and place, re-create the macro-level system by resuming interacting and re-engaging the associated social practices and meanings. As participants within the larger community engage these social practices and meanings in micro-level complex systems, and do so recurrently, the macro-level complex system is sustained over time and space. Like micro-level social systems, then, macro-level social systems are continually re-emerging across multiple episodes of interacting. Like micro-level systems, macro-level social systems are organized from within, in interacting. They are, in short, continually being “interactively organized” (Arundale, 2020, pp. 26–8, 183, 190–6).

Heritage (2008, p. 312) provides another perspective in arguing that everyday micro-level interacting is itself the primary social institution, given that the core, and very likely universal social practices of turn-taking, of action formation, and of repair are fundamental to all human interacting. Clearly the primary social institution of everyday interacting is sustained across time and space only in the recurrent engaging of the full range of social practices and meanings that characterize everyday interacting, which entails that macro-level social institutions of all other kinds and sizes must likewise be sustained in the recurrent engaging of the social practices and meanings associated with those institutions: a position fully in keeping with Schegloff’s (2006, p. 70) argument that everyday interacting is “the infrastructure for social institutions, the natural ecological niche for language, and the arena in which culture is enacted.”

Yet this re-conceptualization of the emergence of macro-level social systems raises an important question: if macro-level complex systems are interactively organized in recurrent interacting among participants in micro-level complex systems, are the emergent properties of macro-level systems thereby reduced to the emergent properties of micro-level systems? Levinson (2005) argues that “interactional reductionism” is a problematic conceptualization of language and culture. Sawyer (2005, pp. 201–5) argues similarly with regard to social institutions in general, and is more specific in noting that a methodological individualist who attempted to reduce “emergent group properties to the time-course sequence of successive individual acts … would necessarily require a sophisticated interaction analysis of the symbolic meanings of each act; their successive coherence and relevance; and how they are interpreted and taken up by other participants” (Sawyer, 2012, p. 273). As is apparent in the prior two sections, the CCMC, grounded as it is in CA, offers precisely that “sophisticated interaction analysis” of the “time-course sequence of successive individual acts” in everyday human communicating, and in so doing reveals the emergent properties that define micro-level complex social systems. If it were the case that the emergent properties of macro-level complex systems were identical to the emergent properties of micro-level complex systems, then indeed the account of macro-level systems offered here would amount to interactional reductionism. One need ask, then, if there are emergent properties of the macro-level complex systems that are social institutions which are distinct from the emergent properties that define micro-level complex systems?

The answer is clearly “Yes.” Sawyer’s (2005) careful review of the literature on social emergence makes evident that sociologists have always argued that macro-level social institutions exhibit order and characteristics not observed in individuals, or in micro-level groups. Sawyer (2005, pp. 94–7) indicates as well that since the advent of general systems concepts in the mid 20th century, sociologists have observed that macro-level social institutions exhibit all four generic emergent properties that distinguish complex systems from additive collectivities: non-aggregativity, non-decomposibility, non-localizabilty, and complexity.

From the perspective of the re-conceptualization offered here, *the central emergent properties defining a macro-level social institution are the operative interpretings of action and meaning formed in micro-level interacting, together with the commonality both in those interpretings and in the social practices, that the participants associate with that macro-level system, and that they maintain in recurrent everyday micro-level interacting over time and space.* CA research across a range of institutions makes apparent that although participants orient to differences between everyday interacting and institutional interacting, there is little evidence that particular institutions have unique social practices. Instead, interaction in institutions is characterized not only by particular social actions and meanings, but also by particular subsets of the broad range of social practices engaged in everyday interacting, and in some cases by particular variations of those social practices, as for example in teacher questioning in educational institutions (Heritage and Clayman, 2010, p. 17). As in Section 4.2, commonality across participants both in their interpretings of the social actions and meanings, and in the social practices associated with a particular social institution, must also emerge if that social institution is to be sustained over time and space.

Beyond these central emergent properties are several others specific to the macro-level systems that participants form as they engage in everyday interacting. Again, brief descriptions of four such properties must suffice.

1. As one aspect of relating, *operative interpretings of membership categorization* emerge regularly in everyday interacting (Pomerantz and Mandelbaum, 2005; Schegloff, 2007a), but are a particularly important property of macro-level social institutions. As participants engage practices in the domain of membership categorization in designing and interpreting utterances, in conjointly co-constituting operative interpretings of action and meaning in micro-level interacting, they position themselves as professionals and clients, or as service providers and customers, for example. A macro-level social system defined in part by such categories emerges across time and space in multiple episodes of such positioning in micro-level interacting.
2. The emergent creating, sustaining, and changing of *sociality*, understood here as participation in a macro-level system like a team, institution, or cultural group (cf. Arundale, 2020, p. 203). Participation in a particular macro-level social system requires being able to engage those social practices and meanings for which the participants have already established commonality. As participants recurrently engage or refrain from engaging these particular practices and meanings in micro-level interacting, or conjointly co-constitute operative interpretings that are consistent or inconsistent with their projections, they identify who is and who is not a participant in that system, and in so doing create, sustain, or change the boundaries of the macro-level social system (Arundale, 2020, p. 195; Krippendorff, 2009, pp. 226–7).
3. The emergent *diversity within macro-level systems*. Within larger macro-level social systems there may well be temporal, spatial, and/or energy limits on the ability of participants to interact with others, leading to more frequent recurrent conjoint co-constituting within smaller local groupings of participants, and potentially to differences in social practices and meanings between those local groupings (cf. Goodwin, 2018, pp. 455, 475). Such diversity is commonly observed in studies of complex systems, as for example in the emergence over time of sub-cultural groups (Arundale, 2020, p. 197–8).
4. The emergent *stability of macro-level systems*. As participants interactively organize macro-level systems they create what Krippendorff (2009, Chap. 18) identifies as temporal, associative, and structural “social memory,” commonality being one example of structural social memory. All three forms of social memory are emergent and distributed across the participants in a macro-level system in their recurrent conjoint co-constituting, not lodged in the participant’s personal memories. Social memory is a key component of the emergent stability of an institution or cultural group, together with the array of social practices through which participants acknowledge departures from a social practice in ways that nevertheless sustain that practice (Arundale, 2020, pp. 199–202, 227).

Each of these specific emergent properties serves to define a larger community of participants as complex, macro-level social system. None of these are properties of the participants as individuals. All of these properties are created, sustained, and changed only in interacting. All are interactively organized, and comprise different facets of *the emergence of macro-level social order in recurrent everyday micro-level interacting.* As Gibbs and Van Orden (2010, p. 162, cf. Craig and Tracy, 2021, p. 160) argue, the structure of a complex system is “not imposed from outside forces or from internal blueprints.” A system’s emergent properties are “temporary, or ‘soft-assembled,’ because they go away when a dynamic linkage changes sufficiently; they have no separate off-line or dormant status in the components of a system.” From the perspective of the re-conceptualization offered here, the macro-level complex systems that are social institutions and cultural groups are characterized by emergent properties that are distinct from the emergent properties of the micro-level complex systems that are essential to creating, sustaining, and changing micro-level systems. Accordingly, the CCMC offers what I identify as a “non-reductive interactionist” account of the emergence of social order at both the micro- and macro-level in everyday interacting (Arundale, 2020, pp. 209–15), not an interactional reductionist, nor a methodological individualist account.

One added observation is in order regarding this re-conceptualization of the emergence of macro-level social order. Because the accounts offered here, both of micro-level order and in turn of macro-level order, rest on participant’s use of social practices in interacting, they may appear to be restricted to everyday face-to-face situations, ignoring situations in which participants use artifacts like ATMs for banking or laptops for grocery shopping. Human beings have constructed a wide array material and energetic artifacts that they engage in everyday interacting, and that according to some accounts (e.g., LaTour, 2005; Cooren, 2010) exhibit agency just as do human beings. In view of the assumptive commitments that underlie the CCMC, however, the phenomena that such accounts treat as agency are manifest only as human agents, defined as in Section 4.1, engage social practices in interacting that employ or involve such artifacts (Arundale, 2020, pp. 230–1). Goodwin (2018), Nevile et al. (2015), and Suchman (2007) all provide penetrating accounts, informed by CA and by ethnomethodology, of how human agents engage artifacts in their everyday and professional interacting.

### 4.4 Researching the emergence of macro-level social order in everyday interacting

The re-conceptualization of the emergence of macro-level social order offered in Section 4.3 begs further exploration, as well as empirical evidence, and providing both presents researchers with unique challenges. Research in CA has already provided important insights into how communicating among two or a few participants is fundamental in the emergence of the properties that define larger, complex social systems like organizations (e.g., Boden, 1994; Heritage, 2005; Heritage and Clayman, 2010), markets (Heath, 2013), professions (Goodwin, 2018, Part V), and more (Arundale, 2020, p. 220). But because the properties of the macro-level social order that emerge in recurrent interacting are conjointly co-constituted across both time and space, it is likely that research methods in addition to CA will be necessary in exploring and grounding the re-conceptualization. Recent research in what has become known as longitudinal CA has begun to reveal how social practices and meanings emerge and come into use as groups of individuals interact over time in extended families (Beach, 2009) and in theatre ensembles (Deppermann and Schmidt, 2021; Schmidt and Deppermann, 2023). Deppermann and Streeck’s (2018), Pekarek Doehler and Deppermann’s (2021), and Pekarek Doehler et al.’s (2018) edited collections include a wide range of longitudinal studies employing CA. Studies such as these indicate that larger social systems are realized episodically over time, and offer important insights into the emergent properties that define such systems, although they were not designed specifically to examine such properties. In commenting on the importance of longitudinal CA, Deppermann and Pekarek Doehler (2021, p. 138) argue that the “detailed analysis of the microlevel organization of social interaction, which is the hallmark of CA … can also send light on larger scale social orders,” and they provide a broad, “integrative picture” of how such orders emerge over time in recurrent interaction among individuals—a picture entirely consistent with the re-conceptualization of the emergence of social order offered here.

Research informed by CA methods such as that sketched above will remain particularly important, but other methods may be useful as well, if like CA they are capable of providing evidence of emergent properties. In general terms, exploring the re-conceptualization and providing empirical evidence will require new research and new research methods that directly address the question: How do the properties that define macro-level social systems emerge over time and space in recurrent interacting in micro-level social systems? More specifically, because the re-conceptualization of macro-level emergence rests on the conceptual framework of the CCMC, new research and new research methods must provide evidence of the conjoint co-constituting of operative interpretings in everyday interacting. Krippendorff (1970) argues that a researcher’s conceptualization of the phenomenon under study provides the framework for all procedures in the conduct of inquiry: (a) making observations, (b) generating data, (c) analyzing those data to produce evidence, and (d) using the evidence in interpreting the outcomes with respect to the conceptualization. These four procedures are tightly linked, such that producing evidence capable of warranting emergent properties places clear demands on the nature of the data a researcher must generate. I examine the requirements for all four procedures in research that engages the CCMC (Arundale, 2020, pp. 362–71), leading to a set of seven requirements that need to be met if a given method is to provide the necessary evidence of emergence in conjointly co-constituting interpretings.

Research using CA methods, as examined in the contributions to Sidnell and Stivers’ (2013) *Handbook*, and as described in textbooks on CA, meets all seven requirements, with longitudinal CA being especially relevant. Space allows only brief indications of seven other methods that may be useful as well in research seeking to probe and ground this re-conceptualization of the emergence of macro-level properties. Edwards (2005) indicates that research in *discursive psychology* has drawn increasingly on CA, and where it does so the methods engaged address the seven requirements. When employed in a manner consistent with CA, as in Fitzgerald (2015) and Schegloff (2007a), *membership categorization analysis* should also meet the requirements. Research informed by *ethnography of communication* (Carbaugh, 2005), and by Craig and Tracy’s (2021) *grounded practical theory*, may draw on CA, discursive psychology, and/or membership categorization analysis, and again, where they do so they may address several of the requirements, though may fall short of providing the needed evidence of participant operative interpretings. Tracy (2010), for example, employs grounded practical theory in a longitudinal study of school board meetings, identifying key social practices that characterize “ordinary democracy.” Two recent arguments that *CA-informed formal coding* (Stivers, 2015) and *experimental and laboratory methods* (Kendrick, 2017) both have been, and will remain useful in addressing issues in CA research, will very likely spur the development of these methodologies, as well as of new combinations of methods for studying everyday interacting. In all cases the extent to which a study addresses the seven requirements for evidence of emergence of macro-level properties can be only assessed by examining its particular research design.

Lastly, while *agent-based modeling* can potentially address most of the seven requirements for methods, and can model large numbers of agents interacting with one another over time, there are challenges in using it in studying the emergent properties of the macro-level social systems formed and maintained in everyday interacting. In closing his book, Sawyer (2005, p. 230) argues that the best way to examine how the properties of macro-level social systems emerge “is to combine the empirical study of socially embedded communication with richly constructed artificial society models.” CA provides the “empirical study of embedded of socially embedded communication” as the first element in this research program, and agent-based modeling (ABM) provides the basis for developing “richly constructed artificial society models” as the second element (cf. Arundale, 2020, pp. 183–90). ABM is one instance of a relatively new research methodology that Poole et al. (2002, p. 31) identify as “modeling inquiry” in which the normally separate procedures of theorizing and generating data for analysis are merged into the single process: the procedural implementation of a theory or model in the simulation generates the data to be analyzed in refining or testing the theory or model.

Very briefly, ABM requires a researcher to model (1) a set of autonomous agents (e.g., persons) with particular attributes and behaviors, (2) a set of procedures that define how and with whom these agents may connect, and (3) potentially an environment with which the agents may interface (Macal and North, 2010, p. 152). Once a researcher has specified protocols for the agents and for their connecting, together with an environment, he or she implements a simulation, often as a computer program, in which each agent connects with another agent, carries out its connecting protocol, processes what it receives, changes its states accordingly, and generates outputs for other agents. Agent-based simulation proceeds episodically: once one episode of connection terminates, the agent begins a new episode by establishing a new connection with another agent: there would be no system whatsoever apart from agents connecting with other agents. Central to research using ABM is observing the changes in the states of the agents and of the system as a whole as the simulation progresses in time, and it is these data that enable the researcher to identify emergent properties that appear as the simulation progresses. Gilbert (2020), Macal and North (2010), and Sawyer (2005) review a wide range of agent-based simulations that exhibit emergent properties such as diversity and stability (Arundale, 2020, p. 187).^8^ Again Sawyer (2005, pp. 22–3, 187) notes that one challenge in employing ABM in studying emergence in macro-level social systems is the absence of a “sophisticated” account of human communication that specifies how one agent connects with another agent—a challenge addressed directly by the CCMC (Arundale, 2020, pp. 381–2; Appendix 2 and 3). A far more important challenge, is formulating viable proxies in a computer simulation for human interpretings of action and meaning. ABM will not replace research methods informed by CA, but it is a relevant method given its potential in discovering new emergent properties and in providing evidence of identified emergent properties of the macro-level social systems that participants create and maintain in recurrent conjoint co-constituting in large communities of human agents.

Employing any of these research methods in studying human interacting is subject to all of the ethical concerns surrounding inquiry regarding human beings. Drawing on Krippendorff’s (2009, Chaps. 1, 6) insightful analyses, I examine the ethical issues involved in modeling, theorizing, comparing conceptual frameworks, and conducting inquiry employing the CCMC (Arundale, 2020, pp. 233–7, 371–60), as well as the CCMC’s implications for ethical personal conduct in interacting (Arundale, 2020, p. 350–3). These ethical issues must be addressed because communicating is central to who we are as persons and as communities. How we come to understand communicating in our theorizing, how we carry out our research on it, and how we engage in everyday interacting in light of those understandings will come to touch the persons we theorize about, the persons who participate in our research, and the persons around us, inclusive of the theorist, the researcher, and ourselves.

## 5 Discussion: re-conceptualizing a venerable sociological concept

Social theorists have long puzzled over how macro-level social order is linked to the micro-level activities of individuals, and in addressing that puzzlement have offered various accounts of how social institutions arise in everyday relations among individuals. This chapter continues in that tradition, acknowledging Sawyer’s (2005) account in terms of interactional frames, but acknowledging as well Rawl’s and Garfinkel’s arguments that accounts of the interaction order in terms of concrete social practices are more productive than accounts in terms of conceptual typifications like frames. The Conjoint Co-constituting Model of Communicating offers an account of how participants use social practices in forming operative interpretings of meaning and social action across triadic sequences of utterances in everyday talk and conduct. Operative interpretings of meaning and social action are emergent (non-linear, non-additive) properties that define micro-level complex systems of two or a few persons. Persons are able to form operative interpretings of action and meaning in everyday interacting with multiple other persons in larger communities because in using the social practices needed to form operative interpretings, in recurrent micro-level interacting over time and across space, they maintain commonality in those practices with those other persons, and so maintain the community. If the social practices are within the domains of epistemics, or of deontics, for example (Stevanovic and Peräkylä, 2014), then the community is engaging and maintaining its normative social order for the distribution of knowledge, or of power, among its members. If the social practices are the universal practices of turn-taking, action formation, and repair, then the community is what Heritage (2008) identifies as the “primary social institution” of everyday interacting. If the social practices and meanings are those associated with money, or instruction, for example, then the community is a financial or an education institution. If the social practices and meanings are those for concatenating vocalizations into words and words into utterances, then the community is a language group. And if the social practices and meanings are those associated with beliefs or kinship relations, for example, then the community is a cultural group. Again, the central emergent properties defining a macro-level social institution are the operative interpretings of action and meaning formed in micro-level interacting, together with the commonality both in those interpretings and in the social practices, that the participants associate with that macro-level system, and that they maintain in recurrent everyday micro-level interacting over time and space.

Re-conceptualizing the emergence of macro-level social order in view of a new conceptualization of the emergence of micro-level social order not only offers the “full-fledged, processual-dynamic view of social emergence” that Sawyer (2005, p. 115–6) finds missing in Durkheim and subsequent theorists, but also addresses sociology’s persistent questions regarding “How is order at the macro social level related to order at the micro individual level?” or “What is the relationship between what is social and what is individual in human life?” Given that everyday interacting among individuals is a universal social phenomenon, altogether fundamental to our nature as human beings, it follows that the account offered here of the emergence of micro-level social order, and in turn of the emergence of macro-level social order in everyday interacting, is an account responsive to Durkheim’s ([1895]1964, p. 98) quest for an explanation of how “collective life … emanates from human nature in general.”

## Ethics statement

Ethical approval was not required for the study involving humans in accordance with the local legislation and institutional requirements. Written informed consent to participate in this study was not required from the participants or the participants’ legal guardians/next of kin in accordance with the national legislation and the institutional requirements.

## Author contributions

The author confirms being the sole contributor of this work and has approved it for publication.

## Footnotes

^1^Two notes regarding terms in the title. First, I use the progressive “interacting,” rather than “interaction,” both in the title and elsewhere, following Pollner’s (1979, p. 253n11) observation that, “[t]o attend to the –ing of things involves a radical modification of the attitude of daily life, for it requires attending to the processes of constituting in lieu of the product thus constituted.” I will later use “communicating” rather than “communication” for the same reason. Second, I identify “emergence” as a “vernerable sociological concept” in the same sense Corning (2002) adopts in his article “The Re-emergence of ‘Emergence’: A Venerable Concept in Search of a Theory.” Corning traces the history of accounts of emergence in research on evolution in biology, dating back to Mill, ([1843]1872) with roots in Aristotle’s *Metaphysics*. Corning’s study of emergence and his alternative approach are highly informative, and although not directly relevant to the argument here, make apparent that “emergence” is a well-worn concept in biology, just as in sociology.

^2^The terms “micro” and “macro” have numerous definitions in the sociological literature (e.g., Münch and Smelser, 1987). In what follows, these terms are understood in their concrete sense, with “micro-level” referring to encounters and interaction among two or a few human individuals (*cf.* Verhoeven, 1985, p. 87), whether face-to-face or mediated, and “macro-level” referring to larger, multi-individual groups, organizations, institutions, and cultures distributed over both time and space.

^3^Sawyer’s references to “complex systems,” “complex systems theory,” and “societies as complex systems” bear no relationship to the various conceptualizations of or to the body of research on “language as a complex adaptive system” (Bechner et al., 2009). “Languaging,” understood as language use in interacting, clearly occurs within complex systems, but Sawyer focuses on the emergence of macro social order, not on language or language use. Similarly, this chapter’s re-conceptualizing of emergence in complex systems has only tenuous links with research in the broad “enactive approach” to understanding “mind and language in social interaction” (DiPaolo et al., 2018), although DeJaegher et al. (2016) argue for bridging the current, rather considerable divide between the understandings of interaction in the enaction approach and in Conversation Analysis.

^4^The practices of overall structural organization are also widespread, although studies of practices like opening telephone conversations, for example, show cultural variation (Hopper, 1992). Human beings have employed practices for opening and closing face-to-face conversations for millennia, and have adapted these practices to address new the contingencies that have arisen, for example, with the invention of the telephone, and much more recently with the development of cell phone technology.

^5^This excerpt is a slightly simplified version of part of a longer conversation presented in Beach (1996, pp. 114-25). The conversation was audio-recorded only, so that details such as body posture and gaze are not available. The audio recording may be obtained for teaching and research purposes only by contacting the current author. Dr. Beach’s generosity in making the recording available for these purposes is gratefully acknowledged. His extended analysis rewards a careful reading.

^6^Lerner and Raymond (2021) provide an alternative to Schegloff’s characterization of the default organizing principle that is more readily applicable in examining elements at finer levels of granularity. They argue that the structural projectability of action and its progressive realization are conjoined and interdependent operations: “First, as actions are launched in their sequential environment, they can be recognized as a possible particular action-in-progress and thus project what more there is to come as the action develops. Then as the action progresses, its further development is inspected to see if it is the progressive realization of the projected action, a change in that action, or its abandonment. Finally, this progressive realization of the action-so-far informs its further projection (as continuing the action or not)” (2021, 279–80). Lerner and Raymond provide evidence for the structural projectability/progressive realization of action at the level of micro-momentary hand movements that interfere with evolving manual action in everyday interacting.

^7^As they conjointly co-constitute a sequence of interaction, participants face difficulties in forming interpretings if they do not share one or more of the social practices being presumed, most obviously those in the domain of language structure, but those in other domains as well. In these cases the participants many find recourse in the universal practices of conversational repair.

^8^In Arundale (2020) I introduce agent-based modeling of complex systems following a brief sketch of social network analysis (2020, pp. 184–6), which has uniformly treated social networks as additive collectivities, not as non-additive, complex systems. As a matter of clarification, then, the re- conceptualization of emergence in macro-level complex social systems that I offer here bears no relationship either to social network analysis in general, or to LaTour’s (2005) Actor-Network Theory in particular.
